# Microstructural changes of white matter fiber tracts induced by insular glioma revealed by tract-based spatial statistics and automatic fiber quantification

**DOI:** 10.1038/s41598-022-06634-5

**Published:** 2022-02-17

**Authors:** Xiangdong Wang, Chunyao Zhou, Yinyan Wang, Lei Wang

**Affiliations:** 1 Beijing Neurosurgical Institute, Capital Medical University, Beijing, China; 2 Department of Neurosurgery, Beijing Tiantan Hospital, Capital Medical University, No. 119 West South Fourth Ring Road, Beijing, 100070 China; 3 Department of Neurosurgery, Heji Hospital, Changzhi Medical College, Changzhi City, Shanxi province, China

**Keywords:** CNS cancer, Cancer imaging

## Abstract

Gliomas typically grow along white matter fiber tracts, yet their invasion patterns remain unclear. This study assessed the effect of insular glioma on large white matter fiber tracts and the microstructural subcortical changes associated with clinical outcomes in patients with insular glioma. Twenty-five patients with insular glioma were enrolled and divided into left and right groups according to tumor lateralization. The control group comprised 14 healthy volunteers. Subjects in both the glioma and control groups underwent diffusion tensor magnetic resonance imaging at 3.0 T. The characteristics of white matter fiber bundles were analyzed using tract-based spatial statistics and automatic fiber quantification. Both Automatic Fiber Quantification and Tract-Based Spatial Statistics revealed that patients with insular glioma had significantly lower fractional anisotropy (FA) values in the inferior frontal-occipital fasciculus and uncinate fasciculus ipsilateral to the tumor, than the controls. Fractional anisotropy associated with mean diffusivity values several large fiber tracts showed potential on tumor-grade distinguishing. Diffusion metrics can sensitively detect microstructural changes in tumor progression. Insular glioma significantly affects the microstructure of white matter fibers proximal to the tumor. The range of white matter fiber bundles affected differs according to the grade of the glioma. These changes are mainly associated with early-stage tumor invasion.

## Introduction

The insular lobe is a deep, complex structure surrounded by multiple white matter fiber bundles, which is suspectable to gliomas^1,2^. Insular lobe gliomas grow along white matter fiber bundles, forming complex tumors. Identifying tumor-invasion range before surgery can minimize the complications caused by surgery-related cortical or subcortical damage, including seizure, and motor or cognitive dysfunction^3–5^.

The invasion of glioma into functional areas of the brain may cause dysfunction, and may subsequently damage microstructural subcortical structures and activate white matter reorganization^6–8^. The growth and invasion of a glioma is a relatively slow process, during which both gray and white matter may structurally and functionally damaged or reorganize sites local and distant to the lesion^9^, which may affect the patient’s postoperative recovery. However, the micro-scaled alterations of early-stage invasion and reorganization are difficult to discover using conventional MRI sequences.

Diffusion tensor imaging (DTI) can quantitively measure the structure of intracranial white matter. The post-processed diffusion maps, including the fractional anisotropy (FA) , Mean Diffusivity (MD), Axonal Diffusivity (AD) and Radial Diffusivity (RD) can reflect the status of fiber degeneration or reorganization. It has been reported that DTI has a higher diagnostic sensitivity for early invasion of glioma than does general MRI^10,11^. A macroscale DTI-based glioma study found significant glioma-related changes in many white matter regions in several DTI coefficients, which were sensitive in early-stage tumor progression^12–14^. Latini et al.^15^ demonstrated a link between DTI metrics and early infiltration identified by pathology. Huang et al. found that voxel-level DTI metrics were a sensitive predictor of histopathological subtypes of insular glioma^16^. DTI is a highly sensitive and non-invasive technique to study neuroplasticity of the macroscopic brain connections in vivo^17–20^.

The tract-based spatial statistics (TBSS) analysis technique for whole-brain fiber bundles^21^ enables voxel-level statistical testing of variations in the major white matter pathways of the whole brain. The accuracy and reproducibility of TBSS analyses are better than those of conventional fiber bundle tracking or whole-brain voxel-level analysis. However, TBSS has an obvious limitation. Namely, using a FA skeleton as a mask for group analysis means discarding orientation information captured in diffusion-weighted imaging data. Thus, TBSS is not able to accurately assign FA values to the same white matter tract across subjects in a consistent way^22^. The Automatic Fiber Quantification (AFQ) method can quantitively measure DTI metrics along with fiber tractography, and can analyze microstructural alterations without the loss of orientation information^23^. AFQ can thus overcome the methodological limitations of TBSS and provide detailed information in white matter tracts. Yet, no study has demonstrated associations between insular glioma invasion and global subcortical change. The current study assessed the effects of insular glioma growth on white matter fiber bundles using AFQ and TBSS. We hope the results of this study will help elucidate the mechanisms underlying early invasion in patients with insular glioma.

## Methods

### Statements

The study protocol was approved by our hospital’s institutional review board. All participants provided written informed consent before data acquisition. All methods were performed in accordance with the relevant guidelines and regulations.

### Participants

Thirty patients (15 men, 15 women; age range: 27–66 years, mean ± SD: 44.17 ± 10.42 years) who had been diagnosed with insular glioma in our hospital between 2014 and 2017 were originally recruited. All enrolled patients and controls are right-handed. Of the 30 patients, five were excluded because of contralateral involvement observed on T2-weighted images. Thus, 25 patients (13 men, 12 women; age range: 27–61 years, mean ± SD: 43.23 ± 10.59 years) with either right (n = 12, RIG group) or left (n = 13, LIG group) insular glioma were finally included in the analysis (Table 1). Fourteen age- and sex-matched healthy controls were also enrolled. All subjects underwent conventional MRI and DTI, and basic clinical data of the patients were collected (supplementary table 1). All patients received tumor resection with frontotemporal approach or expanded frontotemporal approach and were diagnosed with WHO grade II-IV glioma based on pathology. The tumors were classified according to Yasagil and Zetner’s classification^2,24^.

**Table 1:** Inter-group comparison of Clinical characters of patients.

	Left(n = 13)	Right(n = 12)	con(n = 14)	*P*
Age(mean ± SE)	42 ± 9.6	43 ± 11.5	45 ± 3.4	0.71^a^
Gender(M/F)	8/5	5/7	7/7	0. 60^b^
Histology(HGG/LGG)	4/9	5/7	/	0.32^b^
Epilepsy(ep/nep)	4/9	4/8	/	0.89
Yasagil’s subtype(type3/type5)	3/10	2/10		0.68^b^
Disease duration(days, mean ± SE)	202 ± 129	80 ± 27.7		0.733^c^
Tumor volume	52 ± 6.9	49 ± 4.8		0.341^c^

	LGG(n = 16)	HGG(n = 9)		*P*
Age(mean ± SE)	41.5 ± 2.7	41.5 ± 3.4		0.81^c^
Gender(M/F)	5/11	5/4		0. 23^b^
Epilepsy(ep/nep)	4/12	4/5	/	0.31
Yasagil’s subtype(type3/type5)	5/11	2/9		0.44^b^
Disease duration(days, mean ± SE)	96 ± 20	227 ± 189		0.37^c^
Tumor volume	48 ± 4.9	55 ± 7.7		0.44c

*HG* glioblastoma and anaplastic glioma(WHO III-IV), *LGG* low grade glioma(WHO I-II).

^a^One-way ANOVA.

^b^Fisher’s exact test.

^c^Unpaired t-test.

### MRI data acquisition

The MRI data acquisition extraction methods are mostly the same as our previous study^11^. A MAGNETOM Prisma 3.0 T scanner (Siemens; Erlangen, Germany) was used to perform conventional anatomical MRI with the following scanning parameters: T1-magnetization prepared-rapid acquisition gradient echo (MP-RAGE) was used to record anatomical images (repetition time, TR: 2300 ms; echo time, TE: 2.3 ms; flip angle: 8°; field of view, FOV: 240 240 mm^2^; voxel size: 1.0 × 1.0 × 1.0 mm^3^; slice number: 192). The tumor location was determined by two board-certified neurosurgeons and confirmed by an experienced neuroradiologist, all of whom were blinded to the patients’ clinical information. For DTI, we used a single-shot, echo-planar imaging sequence with the following scanning parameters: axial slices: 75, resolution: 2.0 × 2.0 × 2.0 mm, TR: 6000 ms, TE: 103 ms, FOV: 230 × 230 mm, 30 different directions, b = 0/1000 s/mm^2^, and EPI factor: 154.

### Tumor region of interest extraction

**Figure 1:**
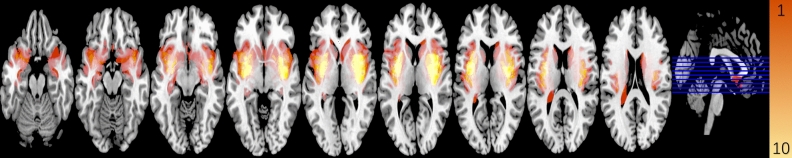
Overlapping map of all tumor lesions. Voxel color indicates the number of overlapping cases from 1 (red) to 10 (yellow).

The Region of Interest(ROI) extraction methods are mostly the same as our previous study^11^. The tumor regions of each patient were manually segmented on T2-weighted images by two experienced neurosurgeons using MRIcron software (https://www.nitrc.org/projects/mricron). Abnormally hyperintense signals in T2-weighted images were determined as tumor areas. The cerebrospinal fluid region was carefully avoided. The final assessment was made by the radiologist. All tumor masks were registered to the MNI-152 standard brain template using the fMRIB software library (FSL; https://fsl.fmrib.ox.ac.uk/fsl/fslwiki/). An overlapping image of either left or right sided gliomas were then generated separately (Fig. 1).

### Tract-based spatial statistics

Data preprocessing and statistical analysis were performed using the pipeline toolbox, PANDA (http://www.nitrc.org/projects/panda/), which was developed based on the FSL. Data processing steps and TBSS analysis were the same as those used by Cui et al.^25^. Briefly, steps to extract basic DTI metrics were implemented, including extracting a brain mask, correcting for eddy current effects, averaging multiple acquisitions, calculating diffusion tensors, and producing metrics. Next, the TBSS process was carried out, which comprised alignment of each subject’s FA image, creating the mean FA map, extracting the FA skeleton, and projecting the individual subject’s FA image onto the skeleton^21^.

### Voxel-wise statistics in TBSS

Voxel-wise analysis was carried out using a general linear model via the FSL randomize tool^26^. General linear model-based voxel-wise regression was applied to identify the correlation between DTI metrics and continuous variables (tumor volume and presurgical disease duration) within each patient group. Inter-group comparisons consisted of a 5000-repetition permutation test between left/right insular glioma groups and healthy controls, respectively. Significant clusters were defined using the threshold-free cluster enhancement method^27^, and the acquired p-value maps were further corrected by the family-wise error rate at the cluster level with *P* < 0.05.

### ROI-based statistics in TBSS

Every fiber mask in the ICBM-DTI-152 white matter atlas was extracted and projected into each individual’s FA and MD map to calculate the mean FA or MD value within the fiber. For every fiber tract’s mean FA and MD, an inter-group unpaired t-test was applied and corrected by the family-wise error rate. To compare difference between LGG and HGG, a left–right “flipping method” was applied before group comparisons in both AFQ and ROI-based TBSS, such that all tumors had the same lateralization. Thus, the regions of interest (ROIs) in every comparison were divided into ipsilateral fibers and contralateral fibers. For every ROI, inter-group comparisons were made according to the variables (e.g. LGG vs. HGG, epilepsy vs. non-epilepsy; t-test, *P* < 0.01). Then, data within significant ROIs were transferred into SPSS to calculate a receiver operating characteristic (ROC) curve; if the area under the ROC curve was > 0.5, the data were transferred to the binary logistic regression model.

### Automatic fiber quantification

An AFQ software package was used for the automatic identification and quantification of subcortical white matter fiber tracts. First, DTI metrics and diffusion tensors preprocessed by FSL were transferred into dt6 formation to fit the AFQ. The AFQ then uses a three-step procedure to identify 18 major fiber tracts in an individual’s brain, as follows: (1) fiber tractography, (2) waypoint ROI-based fiber tract segmentation, and (3) atlas-based fiber refinement. Each fiber group was summarized using a vector of 100 nodes representing the diffusion properties sampled at equidistant locations along the tract. Inter-group differences between Left/Right patients and controls were identified using voxel-wise family-wise error-corrected permutation tests based on FSL randomization (n = 5000). Then, fiber profiles within the left group were “flipped” to re-define ipsilateral fibers and contralateral fibers. The same permutation tests were carried out in those fiber tracts for comparisons between the LGG group and HGG group. Four DTI metrics (FA, MD, Axonal Diffusivity, and Radial Diffusivity) were selected for group comparison and further analysis.

## Results

### Clinical characteristics

The clinical characteristics of all enrolled patients are summarized in Supplementary Table 1. There were 16 patients with LGG (WHO grade II) and 9 patients with HGG (WHO grade III–IV). Five patients were type 3a or 3b, which means purely insular glioma, while others were all type 5, meaning there were frontal or temporal invasion. Five patients (three left, two right) experienced tumor-related cognitive dysfunction, such as memory deterioration, slow reactions, and disorientation. Eight patients experienced tumor-related seizures before surgical treatment. There were no significant differences in age, sex, WHO grade, pre-surgical epilepsy, tumor subtype, tumor volume, presurgical disease duration or deficits in cognitive functioning between the LIGs and RIGs or between HGGs and LGGs (Table 1).

### General ROI-based analysis

**Figure 2:**
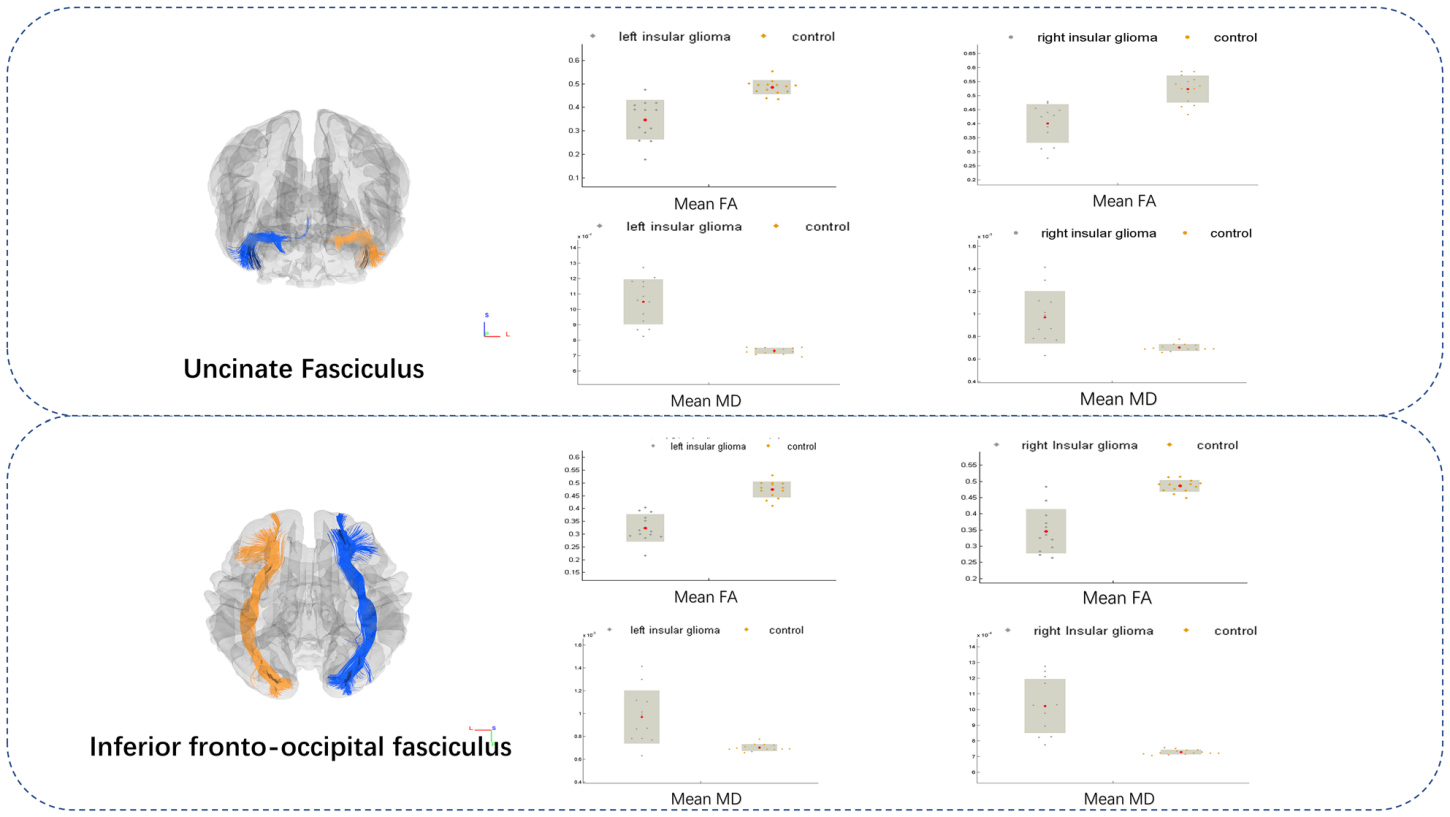
Atlas-based analysis showed significant MD increases and FA decreases at the IFOF and uncinate fasciculus in left- and right-sided glioma patients (two-samples t-test, FWE-corrected *P* < 0.05). A dot-bar figure of group values for MD and FA is shown. Gray dots represent the averaged values of patients, while orange dots represent those of controls.

Significantly decreased FA and elevated MD were observed in the ipsilateral inferior frontal occipital fasciculus(IFOF) and uncinate fasciculus(UF) in both left- and right-sided insular glioma groups (Figs. 2); as expected, these fibers were either within or near the insular lobe. Furthermore, significantly altered fibers in both comparisons (left-sided glioma vs. control, and right-sided glioma vs. control) appeared to be symmetric. However, changes in the right-sided glioma group were more extensive than those in the left-sided glioma group, and a significant change on the sagittal stratum only appeared in the right-sided group (Table 2). No significant FA elevation was observed in the current study.

**Table 2:** Significant fiber tracts in atlas based analysis.

		Left glioma	Control	Corrected *P*^a^
L_IFOF	FA	0.32	0.47	< 0.001
	MD	1.02 × 10^–3^	0.74 × 10^–3^	< 0.001
L_UF	FA	0.34	0.48	< 0.001
	MD	1.04 × 10^–3^	0.73 × 10^–3^	< 0.001
L_SS	FA	0.48	0.52	0.62
	MD	0.87 × 10^–3^	0.78 × 10^–3^	0.11

		Right glioma	Control	Corrected *P*^a^
R_IFOF	FA	0.35	0.48	< 0.001
	MD	1.02 × 10^–3^	0.72 × 10^–3^	< 0.001
R_UF	FA	0.41	0.52	< 0.001
	MD	0.97 × 10^–3^	0.70 × 10^–3^	< 0.001
R_SS	FA	0.48	0.53	0.004
	MD	0.87 × 10^–3^	0.76 × 10^–3^	0.012

*IFOF* Inferior frontal-occipital fasciculus, *UF* uncinate fasciculus, *SS* sagittal stratum.

^a^Student’s t-test, FWE corrected.

**Figure 3:**
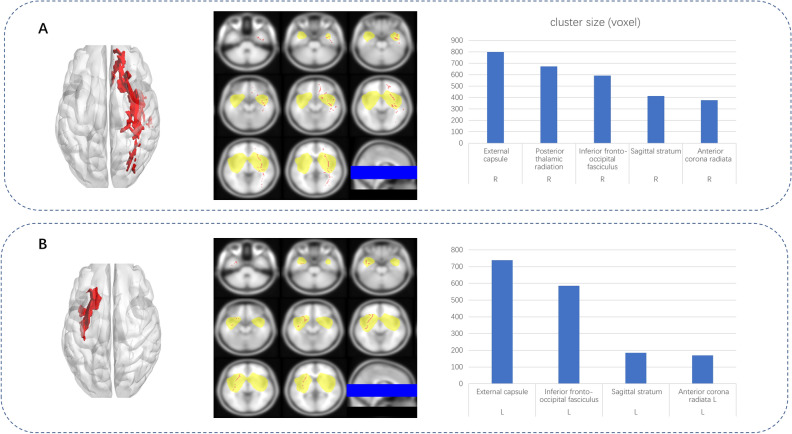
Fiber bundles with different diffusion metrics between the LGG and HGG groups, revealed by atlas-based analysis. A–F: Bar-dot figures showing group difference in six major fiber bundles. G: ROC analysis of all fibers with t < 0.01.

Several fiber ROI showed significant difference between LGG and HGG (Fig. 3). Contralateral external capsule(*P* = 0.009), contralateral internal capsule(*P* = 0.003), contralateral superior frontal-occipital fasciculus(*P* = 0.007), ipsilateral medial lemniscus (*P* = 0.008) and contralateral IFOF in FA and contralateral MD significantly different between the HGG vs. LGG groups, and ROC analysis showed high AUC value out of tumor grade at Contralateral external capsule(AUC = 0.009), contralateral internal capsule(AUC = 0.847), contralateral superior frontal-occipital fasciculus(AUC = 0.833), ipsilateral medial lemniscus (AUC = 0.813) contralateral IFOF(AUC = 0.837) in FA and contralateral IFOF(AUC = 0.837) in MD(Fig. 3). Finally, FA of contralateral internal capsule (*P* = 0.055, Exp(B) = 2.538) and ipsilateral medial lemniscus (*P* = 0.043, Exp(B) = 1.831) were entered into the model in the binary logistic regression analysis (Onward LR) (Table 3).

**Table 3:** Binary logistic regression of averaged diffusion metrics.

		B	S.E	Wald	Significance	Exp(B)
Step 1^a^	Contralateral internal capsule	0.511	0.214	5.698	0.017	1.668
	Constant	− 30.073	12.383	5.898	0.015	0.000
Step 2b	ipsilateral medial lemniscus	0.931	0.485	3.687	0.055	2.538
	Contralateral internal capsule	0.605	0.299	4.088	0.043	1.831
	Constant	− 89.969	40.229	5.002	0.025	0.000

*B* regression coefficient, *S.E* Standard Error, *Exp*(*B*) odds ratio.

### Voxel-based TBSS results

**Figure 4:**
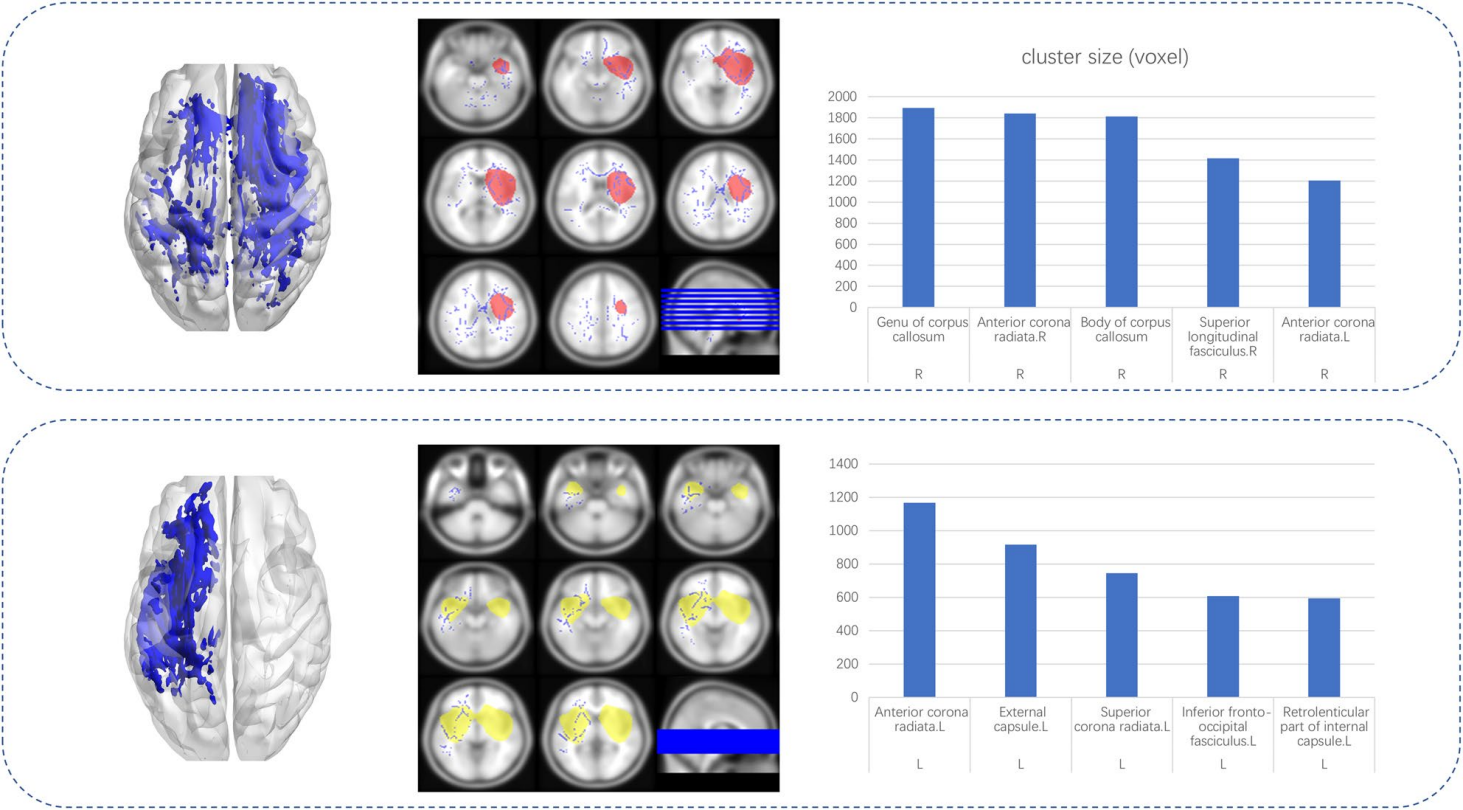
Clusters with significantly lower FA in LIG or RIG compared to the controls, identified by voxel-wise TBSS analysis. A: Left-sided axonal overlapping images of major crossovers (middle), as well as a 3-D rendered image (left). Bar graphs of the most common clusters are shown on the right. B: Right-sided axonal overlapping images of major crossovers (middle), as well as a 3-D rendered image (left). The bar graphs of the most common clusters are shown on the right.

The voxel-wise TBSS analysis revealed significantly reduced major fibers, predominantly above the insular lobe at the tumoral hemisphere, as a result of massive fiber abnormalities caused by tumor presence. (Supplementary table 2 and 3). Widespread clusters of regions of decreased FA were detected in the major white matter tracts (Fig. 4). In LIG group, fibers with FA reduction included external capsule (voxel size: 739), inferior frontal-occipital fasciculus (voxel size: 586), sagittal stratum (voxel size: 185), and anterior corona radiata L (voxel size: 169). In RIG group, fibers with FA reduction included the external capsule (voxel size: 798), posterior thalamic radiation (voxel size: 672), inferior frontal-occipital fasciculus (voxel size: 593), sagittal stratum (voxel size: 414), and anterior corona radiata (voxel size: 377).

**Figure 5:**
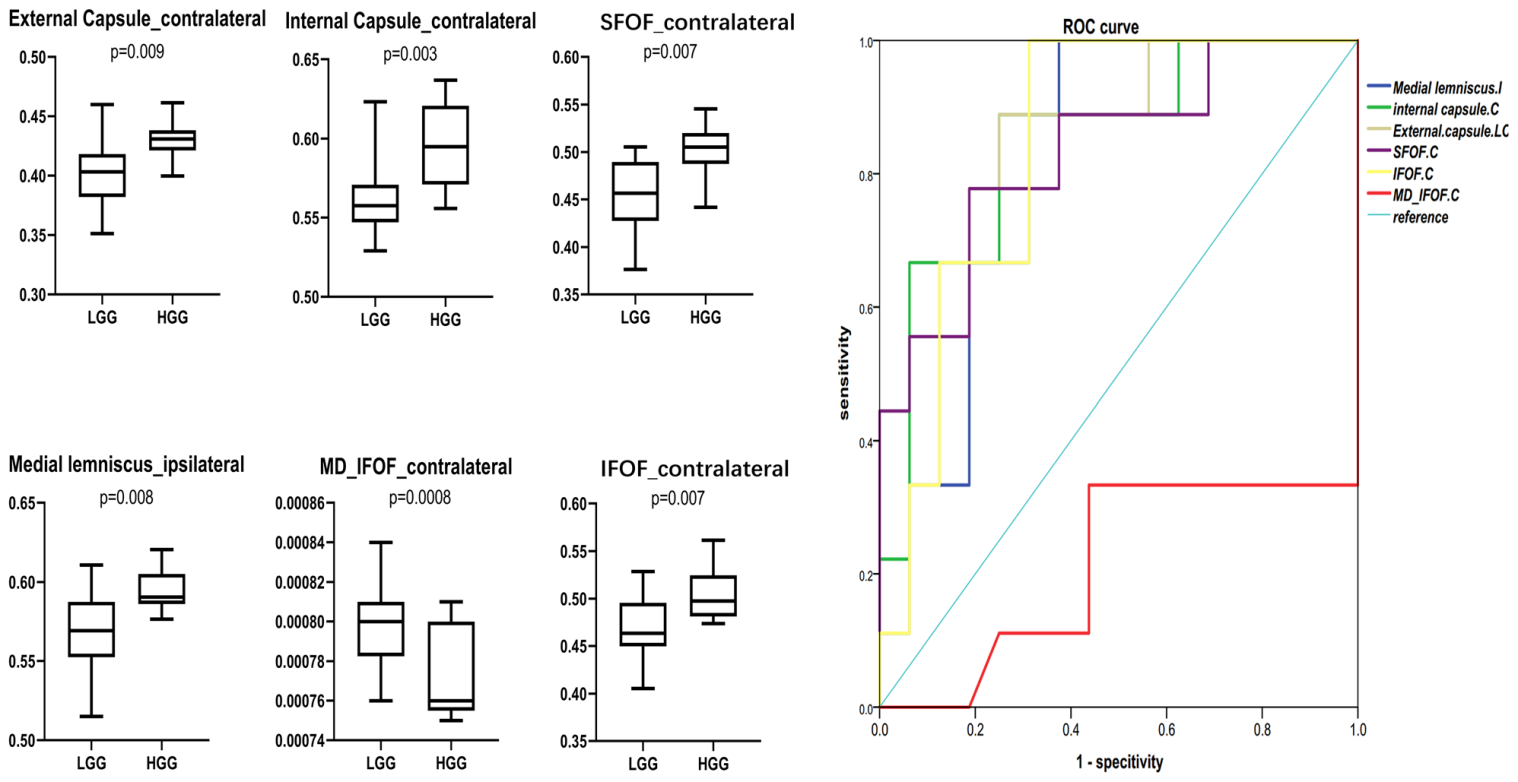
Clusters with significantly higher MD in LIG or RIG compared to the controls, identified by voxel-wise TBSS analysis. A: Left-sided axonal overlapping images of major crossovers (middle), as well as a 3-D rendered image (left). Bar graphs of the most common clusters are shown on the right. B: Right-sided axonal overlapping images of major crossovers (middle), as well as a 3-D rendered image (left). Bar graphs of the most common clusters are shown on the right.

Regions of elevated MD were larger than those of FA degeneration (Fig. 5). In LIG group, the most clusters were found in the anterior corona radiata L (voxel size: 1166), external capsule L (voxel size: 917), superior corona radiata L (voxel size: 744), and inferior frontal-occipital fasciculus L (voxel size: 607). In RIG group, the MD increase was widespread and affected the contra-lesion hemisphere, and essentially involved all major connective fibers. The clusters with the highest voxel counts were located in the genu of the corpus callosum (voxel size: 1894), anterior corona radiata R (voxel size: 1839), and body of the corpus callosum (voxel size: 1814).

No significant clusters or voxels were discovered between FA and tumor volume, MD and tumor volume, FA and presurgical duration or MD and tumor duration in either LIG or RIG group.

### AFQ results

**Figure 6:**
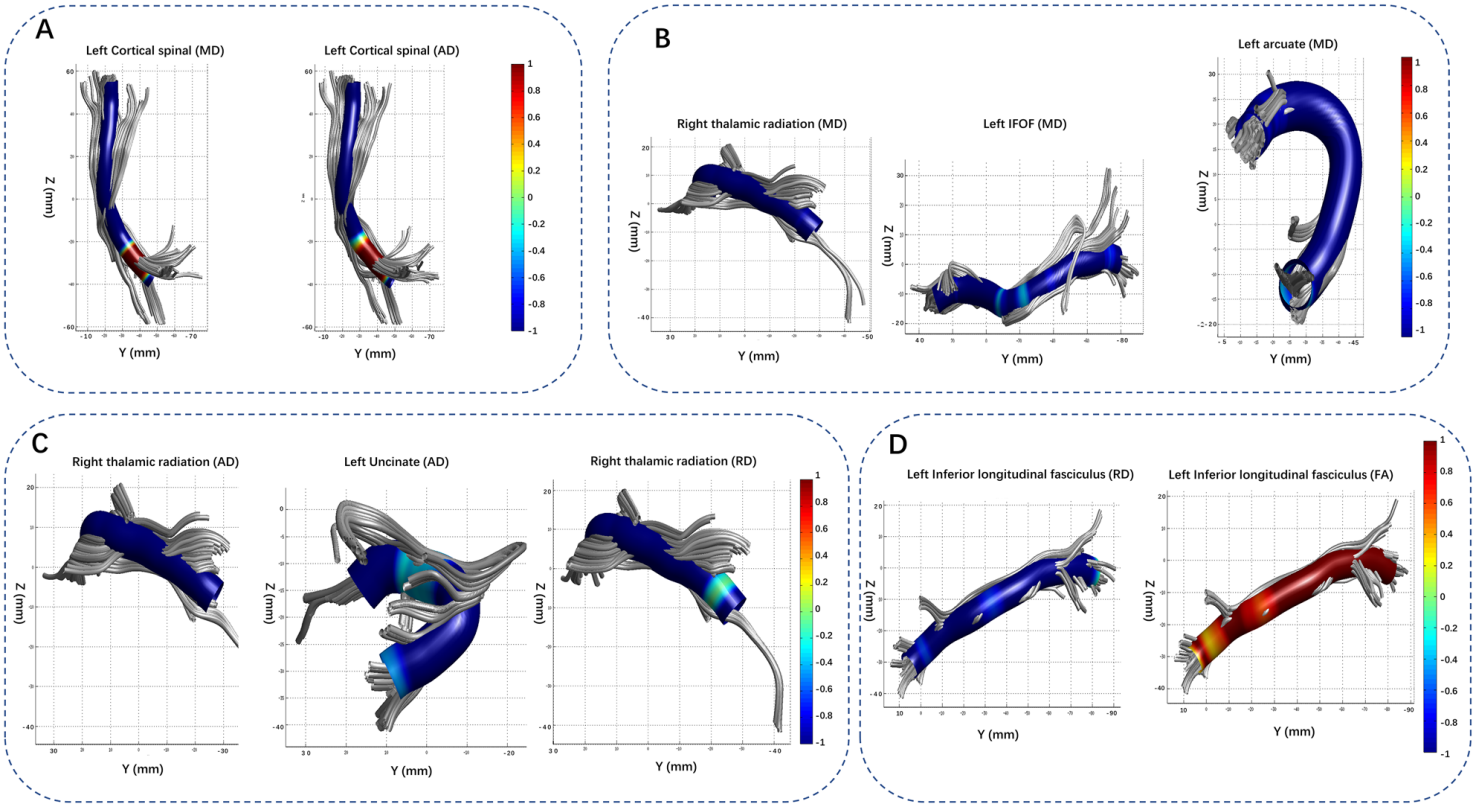
Three-dimensional figures of fiber segmentations with inter-group differences revealed by AFQ. Red-yellow parts indicate an elevation. A: The LIG group vs. control group comparison showed MD elevation in cortical-spinal tract. B-C: The RIG vs. control group comparison showed MD, AD, and RD elevation in the contralateral UF, ILF, and AC. D: Flipped HGG vs. LGG images revealed FA elevation in the contralateral ILF.

AFQ revealed several significant results within control vs LIG, control vs RIG and HGG vs LGG (Fig. 6). When comparing LIG vs control, The MD of segments 86–88 of the second fiber bundle (left corticospinal) were significantly higher, and the AD of segments 47–69 in the second fiber bundle (left corticospinal) was significantly higher. There were no significant differences in the FA or RD.

When comparing RIG vs control, The MDs of the second fiber bundle (right thalamic radiation) segments 1–25 and 52–71, sixth fiber bundle (left inferior frontal-occipital fasciculus) segments 28–32, 76–78, and 95–97, ninth fiber bundle (right superior longitudinal fasciculus) segments 6–10, tenth fiber bundle (left uncinate) segments 99–100, and eleventh fiber bundle (left arcuate) segments 60–68 and 72–77 were significantly higher in RIG. Meanwhile, The AD of the right thalamic radiation segments 2–25 and 65–81, third fiber bundle (left corticospinal) segments 68–80, and tenth fiber bundle (left uncinate) segments 34–38 R index were significantly higher in the RIG. The RDs of the second fiber bundle (right thalamic radiation) segments 3–22 and 56–57, and the ninth fiber bundle (right Superior Longitudinal Fasciculus, SLF) segments 7–9 were significantly higher in RIG.

When comparing the LGG and HGG groups, the FA values for segments 28–32 of the seventh fiber bundle (left Inferior Longitudinal Fasciculus, ILF) were significantly higher in the HGG group than in the LGG group. There were no significant differences in MD and AD. In segments 25–37 of the seventh fiber bundle (left ILF), the RD was significantly lower in the HGG group.

## Discussion

In this study, we used TBSS to assess differences in the characteristics of cerebral white matter fiber bundles between patients with insular glioma and normal controls. This approach revealed an influence of insular glioma on the cerebral white matter fiber bundle skeleton and the plasticity of white matter fiber bundle changes caused by insular glioma.

Insular glioma is a common tumor type in the limbic system. Due to the unique anatomical location of the insular lobe, the relationship of insular glioma to surrounding fiber bundles is more complicated than that of general intracranial tumors. Using traditional imaging data, previous studies have focused on qualitative analyses of the fiber bundles that may be involved in insular tumors^13–15^. These studies could only assess changes in the surrounding fibers due to invasion or edema, which could not be remodeled to preserve local function; such plastic changes in white matter typically occur distal to the tumor.

The current study used TBSS associated with AFQ to analyze microstructural white matter changes associated with insular glioma. This follows on from several previous studies that have reported the relationship between diffusion data and tumor progression^10,14,28^. Diffusion metrics are known to be sensitive markers of early-stage infiltration. In recent years, this field has gradually shifted from exploring the relationship between macroscopic, whole-brain diffusion tensor changes and tumor progression, to exploring the relationship between local, microscopic fiber bundle changes and local tumor invasion. Previous work has revealed that voxels with higher RD and lower FA are associated with glioma cell infiltration^15^. Among the existing studies, only one has focused on insular glioma. Namely, Huang et al. found that FA and MD within the tumor ROI can predict molecular biomarkers^16^; however, a whole-brain fiber bundle-based assessment has been lacking. Thus, microstructural white matter changes revealed by DTI associated with insular glioma remains unclear. The current study has illustrated several pertinent discoveries related to this.

Significant changes in the diffusion coefficient of multiple fiber pathways were detected in insular glioma group. In TBSS, these changes were predominantly distributed among the main fibers proximal to the insular cortex and in fibers connected to the insular cortex. In AFQ, more distant fiber bundle alternation was found. Both analytic methods showed more extensive changes in patients with right-sided glioma. Furthermore, specific diffusion metrics in large fiber tracts showed distinguishing potentials between LGG and HGG.

White matter fiber bundles form the anatomical basis of glioma invasion. Microstructural WM disruptions during glioma invasion was the major focus of the current research. Changes in the diffusion metrics of the WM fiber tracts could primarily be caused by erosion, destruction, impingement, and peritumoral edema of subcortical structure due to tumor infiltration^28^.Moreover, a change in the white matter skeleton diffusion coefficient is also a key parameter that reflects plasticity-associated changes of white matter fiber bundles. As voxel-level diffusion data analysis methods^21^, TBSS associated with AFQ can detect slight changes in whole-brain white matter fiber bundles, and can minimize the influence of non-white matter areas on statistical analyses. The methods used in the current study can be more sensitive and accurate than traditional voxel-level approaches^28,29^.

Changes in diffusion metrics reflect alterations in subcortical white matter fiber bundles and may indicate early-stage tumor invasion The altered fiber bundles could be in, near, or at remote sites from the tumor. Both TBSS and AFQ detected that the inferior frontal-occipital fasciculus and uncinate fasciculus were the main affected fiber bundles. These two fibers were reported as major infiltration pathways of insular glioma. In terms of spatial distribution, the affected white matter was predominantly located in the lower, outer, front, and rear parts of the insular cortex. A previous study focusing on the invasion patterns of insular LGGs suggested that insular gliomas mostly infiltrate alongside long fiber bundles^30^, and it has also been reported that alterations of diffusion metrics could be signs of early invasion, before detection of abnormalities using T2 or FLAIR^10,12^. We believe that these results may be related to the invasion of insular white matter by glioma and could further indicate changes in fiber bundle plasticity. Insular glioma can progress via larger fiber bundles, while distant changes may reflect the reorganization of white matter fibers. Further research is needed to confirm these suggestions.

Different from TBSS, the results of AFQ indicated more distant fiber degeneration. As a matter of fact, the initial automatic fiber tracing algorithm failed to trace fiber bundles influenced by the tumor, thus, only distant fiber bundles were included in the statistical analysis, even though, the results still indicated distant fiber degeneration. The RIG showed much wider range of WM disturbance than the LIG, represented as elevation in MD, AD and RD in several ipsilateral and contralateral fiber bundles. Namely, in the comparison between RIG and control, the disrupted ipsilateral thalamic radiation is both found in TBSS and AFQ, which is a possible indication of posterior tumor extension. Meanwhile, we also found raise in MD and AD of the contralateral IFOF and UF. Interestingly, our TBSS results showed IFOF and UF on the ipsilateral side of tumor is possibly damaged by tumor invasion, and those chronic damage may lead to fiber bundle degeneration at contralateral hemisphere, as reported before^31^.

Interestingly, in the atlas-based analysis, FA values in four major fiber bundles showed the potential to distinguish tumor grade. Furthermore, FA values seemed to be elevated in contralateral fibers within the HGG group. Normally, FA elevation indicates fiber regeneration and reorganization. One study has revealed that slow-growing lesions can cause brain reorganization^9^, and that this more frequently occurs in patients with LGG. An explanation might be that HGG, which causes more severe white matter damage around the tumor, can more strongly activate contralateral white matter plasticity. Further research could focus on this point.

Although nearly the same fiber bundles were involved in both glioma groups in this study, the RIG group exhibited significantly more extensive subcortical damage than the LIG group. Moreover, while there were no significant differences in tumor volume between the two groups, differences in spatial distribution may have resulted in significant differences in distal white matter microstructure. Interestingly, studies have reported that right insular LGG is significantly associated with complex partial epilepsy^32^, while no such reports exist regarding left insular LGG. In a multicenter study involving more than 2000 patients, the mortality and dysfunction rates of patients with acute cerebral hemorrhage involving the right insular lobe were significantly higher than those involving the left insular lobe^33^. Thus, our results suggest that the right cerebral hemisphere is more susceptible to various injuries and/or requires more extensive plastic changes in white matter fiber bundles to compensate for damage.

## Conclusion

In this study, TBSS associated with AFQ was used to quantitatively analyze the white matter changes in large fiber bundles that may be caused by the growth and invasion of insular glioma. The main fiber bundles that exhibited significantly changed diffusion coefficients were the uncinate fasciculus, inferior frontal-occipital fasciculus, and sagittal stratum.

; the coefficient changes included decreased FA and increased MD. These changes in white matter fiber bundles may be precursors of glioma invasion. Furthermore, FA value in several fiber bundles contralateral to the tumor have potentials on distinguishing HGG from LGG.

## Limitations

The current study demonstrated that diffusion metrics in large fiber tracts may be sensitive predictors of tumor infiltration and tumor grade. However, it has several limitations that should be noted. First, as a retrospective study, the number of enrolled patients was small. We were thus obliged to utilize the flipping method to increase the total number for statistical comparisons, which could have sacrificed information concerning the dominant hemisphere and tumor lateralization. Second, the field of quantitative diffusion MRI research now gravitates towards the use of higher-order modeling techniques. Techniques such as constrained spherical deconvolution and diffusion spectrum imaging can address the lack of axonal fiber specificity in DTI. However, due to hardware technological problems, we are currently unable to adopt those techniques. Further research on DTI and insular glioma may focus on these points.

## Supplementary Information

Supplementary Information 1.

Supplementary Information 2.

Supplementary Information 3.

## References

[1] Kucukyuruk B, Yagmurlu K, Tanriover N, Uzan M, Rhoton AL (2014). Microsurgical anatomy of the white matter tracts in hemispherotomy. Neurosurgery.

[2] Yasargil MG (1992). Tumours of the limbic and paralimbic systems. Acta Neurochir. (Wien).

[3] Lang FF (2001). Surgical resection of intrinsic insular tumors: complication avoidance. J. Neurosurg..

[4] Ius T (2014). Surgery for insular low-grade glioma: predictors of postoperative seizure outcome. J. Neurosurg..

[5] Wu AS (2011). Neurocognitive function before and after surgery for insular gliomas. J. Neurosurg..

[6] Hugues D (2005). Lessons from brain mapping in surgery for low-grade glioma: insights into associations between tumour and brain plasticity. Lancet Neurol..

[7] Duffau H (2003). Functional recovery after surgical resection of low grade gliomas in eloquent brain: hypothesis of brain compensation. J. Neurol Neurosurg. Psychiatry.

[8] Bryszewski B (2012). Functional rearrangement of the primary and secondary motor cortex in patients with primary tumors of the central nervous system located in the region of the central sulcus depending on the histopathological type and the size of tumor: examination by means of functional magnetic resonance imaging. Pol. J. Radiol..

[9] Desmurget M, Bonnetblanc F, Duffau H (2007). Contrasting acute and slow-growing lesions: a new door to brain plasticity. Brain.

[10] Kono K (2001). The role of diffusion-weighted imaging in patients with brain tumors. AJNR Am. J. Neuroradiol..

[11] Wang XD, Zhou CY, Wang L, Wang YY, Jiang T (2020). Motor cortex gliomas induces microstructural changes of large fiber tracts revealed by TBSS. Sci. Rep..

[12] Kallenberg K (2013). Glioma infiltration of the corpus callosum: early signs detected by DTI. J. Neurooncol..

[13] Ferda J (2010). Diffusion tensor magnetic resonance imaging of glial brain tumors. Eur. J. Radiol..

[14] Gimenez U (2016). Microscopic DTI accurately identifies early glioma cell migration: correlation with multimodal imaging in a new glioma stem cell model. NMR Biomed..

[15] Latini F (2021). The link between gliomas infiltration and white matter architecture investigated with electron microscopy and diffusion tensor imaging. Neuroimage Clin..

[16] Huang Z (2021). Prediction of lower grade insular glioma molecular pathology using diffusion tensor imaging metric-based histogram parameters. Front. Oncol..

[17] Lerch JP (2017). Studying neuroanatomy using MRI. Nat. Neurosci..

[18] Jbabdi S, Sotiropoulos SN, Haber SN, Van Essen DC, Behrens TE (2015). Measuring macroscopic brain connections in vivo. Nat. Neurosci..

[19] Hofstetter S, Assaf Y (2017). The rapid development of structural plasticity through short water maze training: A DTI study. Neuroimage.

[20] Deng F (2018). Plasticity in deep and superficial white matter: a DTI study in world class gymnasts. Brain Struct. Funct..

[21] Smith SM (2006). Tract-based spatial statistics: voxelwise analysis of multi-subject diffusion data. Neuroimage.

[22] Bach M (2014). Methodological considerations on tract-based spatial statistics (TBSS). Neuroimage.

[23] Yeatman JD, Dougherty RF, Myall NJ, Wandell BA, Feldman HM (2012). Tract profiles of white matter properties: automating fiber-tract quantification. PLoS ONE.

[24] Zentner J, Meyer B, Stangl A, Schramm J (1996). Intrinsic tumors of the insula: a prospective surgical study of 30 patients. J. Neurosurg..

[25] Cui Z, Zhong S, Xu P, He Y, Gong G (2013). PANDA: a pipeline toolbox for analyzing brain diffusion images. Front. Hum. Neurosci..

[26] Nichols TE, Holmes AP (2002). Nonparametric permutation tests for functional neuroimaging: a primer with examples. Hum. Brain Mapp.

[27] Smith SM, Nichols TE (2009). Threshold-free cluster enhancement: addressing problems of smoothing, threshold dependence and localisation in cluster inference. Neuroimage.

[28] Won YI (2016). White matter change revealed by diffusion tensor imaging in gliomas. Brain Tumor Res. Treat..

[29] Schoene-Bake JC (2009). Widespread affections of large fiber tracts in postoperative temporal lobe epilepsy. Neuroimage.

[30] Mandonnet E, Capelle L, Duffau H (2006). Extension of paralimbic low grade gliomas: toward an anatomical classification based on white matter invasion patterns. J. Neurooncol..

[31] Pischiutta F (2018). Single severe traumatic brain injury produces progressive pathology with ongoing contralateral white matter damage one year after injury. Exp. Neurol..

[32] Wang Y (2015). Localizing seizure-susceptible brain regions associated with low-grade gliomas using voxel-based lesion-symptom mapping. Neuro Oncol..

[33] Sposato LA (2016). Effect of right insular involvement on death and functional outcome after acute ischemic stroke in the IST-3 trial (third international stroke trial). Stroke.

