# Supplementary material for: Microstructural changes of white matter fiber tracts induced by insular glioma revealed by tract-based spatial statistics and automatic fiber quantification
Source: Sci Rep. 2022 Feb 17;12:2685. doi: 10.1038/s41598-022-06634-5 (PMC8854665; doi:10.1038/s41598-022-06634-5)
Supplement: Supplementary file 1 — Supplementary Information 1. [file 41598_2022_6634_MOESM1_ESM.docx]

**Table 1.** Summary of patients’ characteristic

| **Patient No.** | **Age** | **Gender** | **WHO grade** | **Tumor Volume(cc)** | **Yasagil’s**  **subtype** | **Involvement** | **Presurgical epilepsy** | **Epilepsy type** | **Duration before diagnosis** | **Postsurgical treatment** | **Cognitive dysfunction** |
| --- | --- | --- | --- | --- | --- | --- | --- | --- | --- | --- | --- |
| L_1 | 33 | M | A(WHO II) | 43.94 | 5A | T | 0 | / | 133 | 1 | N |
| L_2 | 46 | M | GBM(WHO IV) | 106.52 | 5B | F/T | 0 | / | 14 | 2 | Y |
| L_3 | 54 | M | A(WHO II) | 71.97 | 5A | T | 0 | / | 76 | 1 | N |
| L_4 | 34 | M | AA(WHO III) | 56.09 | 5A | T | 1 | Partial | 54 | 2 | N |
| L_5 | 51 | F | AA(WHO III) | 53.79 | 5A | T | 0 | / | 1742 | 2 | N |
| L_6 | 59 | F | A(WHO II) | 15.99 | 3A | / | 0 | / | 95 | 1 | N |
| L_7 | 41 | F | A(WHO II) | 58.3 | 5A | / | 0 | / | 172 | 1 | N |
| L_8 | 34 | F | A(WHO II) | 63.96 | 5B | F | 1 | general | 58 | 1 | Y |
| L_9 | 45 | F | A(WHO II) | 22.89 | 3B | / | 0 | / | 62 | 1 | N |
| L_10 | 34 | F | A(WHO II) | 21.93 | 3B | / | 0 | / | 70 | 0 | N |
| L_11 | 31 | F | A(WHO II) | 55.54 | 5A | F | 1 | general | 94 | 1 | Y |
| L_12 | 38 | F | A(WHO II) | 36.7 | 5A | / | 0 | / | 31 | 1 | N |
| L_13 | 28 | M | AO(WHO III) | 74.3 | 5A | F | 1 | partial | 32 | 2 | N |
| R_1 | 31 | M | GBM(WHO IV) | 40.94 | 5A | F | 1 | general | 31 | 2 | Y |
| R_2 | 53 | F | O(WHO II) | 35.04 | 5A | T | 0 | / | 344 | 1 | N |
| R_3 | 27 | M | O(WHO II) | 46.72 | 5A | T | 0 | / | 172 | 1 | N |
| R_4 | 38 | F | AA(WHO III) | 40.51 | 3B | / | 0 | / | 60 | 2 | N |
| R_5 | 53 | M | GBM(WHO IV) | 38.13 | 5A | T | 0 | / | 16 | 2 | N |
| R_6 | 57 | F | GBM(WHO IV) | 57.90 | 5B | F/T | 0 | / | 67 | 2 | Y |
| R_7 | 61 | F | A(WHO II) | 30.47 | 3B | / | 0 | / | 44 | 1 | N |
| R_8 | 36 | F | GBM(WHO IV) | 31.21 | 5A | T | 1 | general | 30 | 2 | N |
| R_9 | 32 | F | O(WHO II) | 53.08 | 5A | T | 1 | general | 2 | 0 | N |
| R_10 | 32 | M | A(WHO II) | 77.90 | 5B | F/T | 0 | / | 121 | 1 | N |
| R_11 | 55 | M | O(WHO II) | 76.37 | 5A | F | 0 | / | 15 | 1 | N |
| R_12 | 39 | F | A(WHO II) | 66.21 | 5A | F | 1 | general | 60 | 1 | N |

*WHO grades: A:astrocytoma; O:oligodendreoma; AA: anaplastic astrocytoma; AO: anaplastic oligodendreoma; GBM: glioblastoma.*

*Yasagil’s subtype: an classic classification of limbic and paralimbic tumors according to Yasagil. 3A: purely insular glioma. 3B: insular glioma involving* [*operculum*](http://dict.cn/frontal%20operculum)*. 5A: insular glioma involving frontal-orbital lobe or temporal pole. 5B: insular glioma with more than one lobe involvement.*

*Involvement: F: frontal ; T: temporal; F/T: frontal and temporal.*

*Presurgical epilepsy: 0: no seizure history. 1: at least one seizure occurrence before surgery.*

*Postsurgical treatment: 0: no specific treatment. 1: single radiotherapy. 2: combined radio-chemotherapy. (Temozodimide)*
