# Supplementary material for: Microstructural changes of white matter fiber tracts induced by insular glioma revealed by tract-based spatial statistics and automatic fiber quantification
Source: Sci Rep. 2022 Feb 17;12:2685. doi: 10.1038/s41598-022-06634-5 (PMC8854665; doi:10.1038/s41598-022-06634-5)
Supplement: Supplementary file 2 — Supplementary Information 2. [file 41598_2022_6634_MOESM2_ESM.docx]

| **Metric** | **region name** | **voxel size** |
| --- | --- | --- |
| FA | External capsule L | 739 |
|  | Inferior fronto-occipital fasciculus L | 586 |
|  | Sagittal stratum (include inferior longitudinal fasciculus and inferior fronto-occipital fasciculus) L | 185 |
|  | Anterior corona radiata L | 169 |
|  | Anterior limb of internal capsule L | 79 |
|  | Uncinate fasciculus L | 51 |
| MD | Anterior corona radiata L | 1166 |
|  | External capsule L | 917 |
|  | Superior corona radiata L | 749 |
|  | Anterior limb of internal capsule L | 744 |
|  | Inferior fronto-occipital fasciculus L | 607 |
|  | Retrolenticular part of internal capsule L | 593 |
|  | Splenium of corpus callosum | 503 |
|  | Sagittal stratum (include inferior longitidinal fasciculus and inferior fronto-occipital fasciculus) L | 438 |
|  | Posterior limb of internal capsule L | 417 |
|  | Superior longitudinal fasciculus L | 322 |
|  | Posterior thalamic radiation (include optic radiation) L | 281 |
|  | Posterior corona radiata L | 262 |
|  | Fornix (cres) / Stria terminalis (can not be resolved with current resolution) L | 176 |
|  | Genu of corpus callosum | 106 |
|  | Cingulum (hippocampus) L | 92 |
|  | Tapetum L | 82 |
|  | Uncinate fasciculus L | 62 |
|  | Body of corpus callosum | 55 |
|  | Superior fronto-occipital fasciculus (could be a part of anterior internal capsule) L | 50 |

Supplementary Table 2: Significant clusters in control vs left glioma, identified by TBSS.
