# Supplementary material for: Microstructural changes of white matter fiber tracts induced by insular glioma revealed by tract-based spatial statistics and automatic fiber quantification
Source: Sci Rep. 2022 Feb 17;12:2685. doi: 10.1038/s41598-022-06634-5 (PMC8854665; doi:10.1038/s41598-022-06634-5)
Supplement: Supplementary file 3 — Supplementary Information 3. [file 41598_2022_6634_MOESM3_ESM.docx]

Supplementary Table 3: Significant clusters in control vs Right glioma, identified by TBSS.

| **Metric** | **region name** | **voxel size** |
| --- | --- | --- |
| FA | External capsule R | 798 |
|  | Posterior thalamic radiation (include optic radiation) R | 672 |
|  | Inferior fronto-occipital fasciculus R | 593 |
|  | Sagittal stratum (include inferior longitidinal fasciculus and inferior fronto-occipital fasciculus) R | 414 |
|  | Anterior corona radiata R | 377 |
|  | Retrolenticular part of internal capsule R | 353 |
|  | Fornix (cres) / Stria terminalis (can not be resolved with current resolution) R | 215 |
|  | Uncinate fasciculus R | 66 |
| MD | Genu of corpus callosum | 1894 |
|  | Anterior corona radiata R | 1839 |
|  | Body of corpus callosum | 1814 |
|  | Superior longitudinal fasciculus R | 1416 |
|  | Anterior corona radiata L | 1206 |
|  | Splenium of corpus callosum | 1195 |
|  | Superior corona radiata R | 1088 |
|  | Middle cerebellar peduncle | 1087 |
|  | External capsule R | 1019 |
|  | Superior longitudinal fasciculus L | 945 |
|  | Anterior limb of internal capsule R | 907 |
|  | Inferior fronto-occipital fasciculus R | 625 |
|  | Sagittal stratum (include inferior longitidinal fasciculus and inferior fronto-occipital fasciculus) R | 622 |
|  | Retrolenticular part of internal capsule R | 620 |
|  | Posterior limb of internal capsule R | 598 |
|  | Superior corona radiata L | 552 |
|  | Posterior thalamic radiation (include optic radiation) R | 476 |
|  | Posterior corona radiata R | 451 |
|  | Retrolenticular part of internal capsule L | 437 |
|  | Posterior corona radiata L | 369 |
|  | Cingulum (cingulate gyrus) R | 293 |
|  | Anterior limb of internal capsule L | 286 |
|  | External capsule L | 213 |
|  | Fornix (cres) / Stria terminalis (can not be resolved with current resolution) R | 205 |
|  | Inferior fronto-occipital fasciculus L | 195 |
|  | Cingulum (cingulate gyrus) L | 171 |
|  | Pontine crossing tract (a part of MCP) | 167 |
|  | Medial lemniscus L | 132 |
|  | Posterior thalamic radiation (include optic radiation) L | 116 |
|  | Inferior cerebellar peduncle R | 111 |
|  | Medial lemniscus R | 93 |
|  | Tapetum R | 90 |
|  | Cerebral peduncle R | 84 |
|  | Corticospinal tract R | 82 |
|  | Inferior cerebellar peduncle L | 75 |
|  | Superior fronto-occipital fasciculus (could be a part of anterior internal capsule) R | 73 |
|  | Uncinate fasciculus R | 71 |
|  | Sagittal stratum (include inferior longitidinal fasciculus and inferior fronto-occipital fasciculus) L | 56 |
|  | Superior cerebellar peduncle R | 55 |
